# Determination of cut-off cycle threshold values in routine RT–PCR assays to assist differential diagnosis of norovirus in children hospitalized for acute gastroenteritis

**DOI:** 10.1017/S095026881500059X

**Published:** 2015-04-01

**Authors:** N. V. TRANG, M. CHOISY, T. NAKAGOMI, N. T. M. CHINH, Y. H. DOAN, T. YAMASHIRO, J. E. BRYANT, O. NAKAGOMI, D. D. ANH

**Affiliations:** 1 The National Institute of Hygiene and Epidemiology, Hanoi, Vietnam; 2 MIVEGEC (UM1-UM2-CNRS 5290-IRD 224), Centre de Recherche IRD, Montpellier, France; 3 Oxford University Clinical Research Unit, Hanoi, Vietnam; 4 Division of Molecular Epidemiology, Graduate School of Biomedical Science, Nagasaki University, Japan; 5 Thai Binh Paediatric Hospital, Vietnam; 6 Centre for Infectious Disease Research in Asia and Africa, Institute of Tropical Medicine, Nagasaki University, Japan; 7 Vietnam Research Station, J-GRID, Vietnam; 8 The Centre for Tropical Medicine, Nuffield Department of Clinical Medicine, University of Oxford, UK

**Keywords:** Caliciviruses, diarrhoea, estimating disease prevalence, gastrointestinal infections, modelling

## Abstract

Norovirus (NV) is an important cause of acute gastroenteritis in children, but is also frequently detected in asymptomatic children, which complicates the interpretation of NV detection results in both the clinical setting and population prevalence studies. A total of 807 faecal samples from children aged <5 years hospitalized for acute gastroenteritis were collected in Thai Binh, Vietnam, from January 2011 to September 2012. Real-time RT–PCR was used to detect and quantify NV-RNA in clinical samples. A bimodal distribution of cycle threshold (C_t_) values was observed in which the lower peak was assumed to represent cases for which NV was the causal agent of diarrhoea, whereas the higher peak was assumed to represent cases involving an alternative pathogen other than NV. Under these assumptions, we applied finite-mixture modelling to estimate a threshold of C_t_ <21·36 (95% confidence interval 20·29–22·46) to distinguish NV-positive patients for which NV was the likely cause of diarrhoea. We evaluated the validity of the threshold through comparisons with NV antigen ELISA results, and comparisons of C_t_ values in patients co-infected with rotavirus. We conclude that the use of an appropriate cut-off value in the interpretation of NV real-time RT–PCR results may improve differential diagnosis of enteric infections, and could contribute to improved estimates of the burden of NV disease.

## INTRODUCTION

Norovirus (NV) (family Caliciviridae, genus *Norovirus*) is a major cause of gastrointestinal disease worldwide, and the cause of an estimated 200 000 deaths and 1·1 million hospitalizations in children <3 years of age annually [1, 2]. The NV detection rate in diarrhoeal patients varies from as high as 31–48% [3–5] to as low as 3–5% [6], suggesting that the burden of NV disease may be highly variable geographically. Similarly, a wide range of NV prevalence (5–48%) has been observed in different regions of Vietnam [7–10]. While transmission dynamics may indeed vary across regions, differences between studies may also reflect differences in diagnostic methodology. Detection sensitivities depend on many factors such as sample preparation methods, RNA extraction, presence of reverse-transcriptase inhibitors, primer design, amplification chemistries (e.g. enzymes/buffers), virus concentrations in the sample (influenced by the time elapsed from the onset of diarrhoea to sampling, and sample storage conditions), as well as viral factors such as genetic variation in circulating strains. Interpreting NV detection results in both the clinical setting and in population prevalence studies is complicated by the fact that NV is also frequently detected by reverse-transcriptase–polymerase chain reaction (RT–PCR) in asymptomatic individuals [10–17]. Only a few studies have examined and compared the detection of NV between diarrhoeal cases and concurrent non-diarrhoeal controls (reviewed in [18]). In some studies, similar NV detection rates have been observed in both cases and controls, or even higher rates in controls *vs.* diarrhoea cases [12, 19]. NV diarrhoeal patients are known to shed virus for prolonged periods of time following recovery [20].

Shedding of microorganisms without diarrhoeal symptoms is a common phenomenon for many enteric pathogens, such as neonatal rotavirus (RV), bocavirus, NV, *Vibrio cholerae* O1, enterotoxigenic *Escherichia coli*, enteropathogenic *E. coli, Campylobacter jejuni* and *Giardia lamblia* [21–23]. Asymptomatic shedding complicates patient management, studies of disease burden, and monitoring of vaccine trials. Improved methods of inferring a causative role of NV in clinical diagnostics would be a welcome contribution to transmission studies and modelling efforts, with potential applications to clinical diagnostics. Therefore, the objective of this study was to identify a cut-off value for viral load of NV in diarrhoea samples to infer a causative role in diarrhoea.

We started from the observation that distributions of NV C_t_ values in diarrhoeal cases are typically bimodal [19]. We hypothesized that the lower C_t_ value peak corresponds to cases for which the cause of the diarrhoea is NV, and the higher C_t_ value peak corresponds to cases for which another co-infecting agent is the principal cause of diarrhoea. We modelled the NV C_t_ value bimodal distribution with a finite-mixture model, which allowed us to identify a C_t_ threshold value associated with disease risk. We then tested our initial hypothesis using information on RV and NV co-infections as determined by antigen enzyme-linked immunosorbent assay (ELISA).

## METHODS

### Sample collection

The samples and dataset used in this study were generated from a large prospective, hospital-based diarrhoea study in Thai Binh Paediatric Hospital, Thai Binh, Vietnam. Faecal samples of children aged <5 years hospitalized acute gastroenterititis were collected upon obtaining parental consent from 2011 to 2012. Inclusion criteria included diarrhea episode ⩾3 times per 24 h, and admission to hospital within 7 days from onset. The study was approved by the Medical Research Ethical Committee of the National Institute of Hygiene and Epidemiology, Vietnam, and the Internal Review Board of Nagasaki University.

### NV detection and genotyping

For all faecal specimens, 20% suspension in DEPC-treated water was prepared for viral RNA extraction using Qiamp viral RNA extraction kit (Qiagen, Germany). NV genogroups GI and GII were detected as previously described [8, 24, 25], for which the detection limit has been determined as 10–50 RNA copies/reaction. The assay can detect the following different genotypes: GI.1, GI.2, GI.3, GI.4, GI.6, GI.8 and GII.2, GII.3, GII.4, GII.6, GII.7, GII.10, GII.12, GII.13. NV genotyping was performed by amplification of the VP1 gene as described previously [26], followed by sequencing of PCR products or pGEM-T cloned vector products on a 3130xl Genetic Analyzer (Applied Biosystems, USA).

Sequences were manually aligned with BioEdit v. 7.0.5 and submitted to the online Norovirus Genotyping Tool v. 1.0 (www.rivm.nl/mpf/norovirus/typingtool) for genotype determination [27] .

### Evaluation of cut-off cycle threshold for NV

The cycle threshold (C_t_) value from the real time RT–PCR was used as a proxy measure of faecal viral load; C_t_ <40 was considered positive. C_t_ values are inversely proportional (on a logarithmic scale) to viral load, hence lower C_t_ values correspond to higher viral loads. The same positive control was used throughout all experiments, and its C_t_ value varied within 0·5. Threshold level of the thermocycler (0·03) remained constant throughout the analysis.

### Modelling of C_t_ distributions by finite-mixture models

**Fig. 1.**
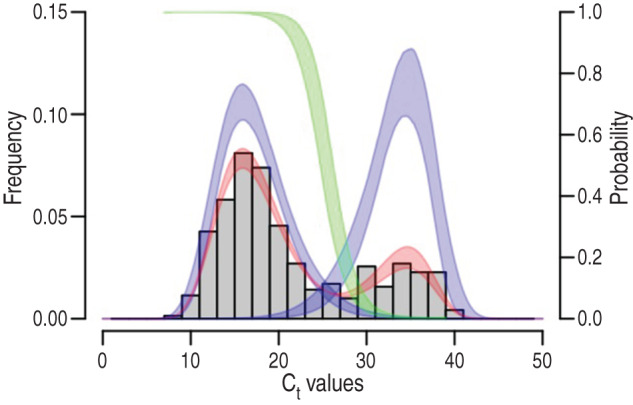
Finite-mixture modelling of the C_t_ value distribution and identification of the cut-off values. The number of samples with C_t_ <40 was 346 and is represented by the grey histograms. The best finite-mixture model was *D*_1_ with a log-normal distribution (left blue curve), and *D*_2_ with a Weibull distribution (right blue curve). This model is shown in red. From these latter two, the probability *P* of belonging to the left-most peak of C_t_ values is computed as *D*_1_**/**(*D*_1_ + *D*_2_) and this is shown by the green curve (see the right vertical scale). The widths of the curves indicate the 95% confidence intervals.

**Fig. 2.**
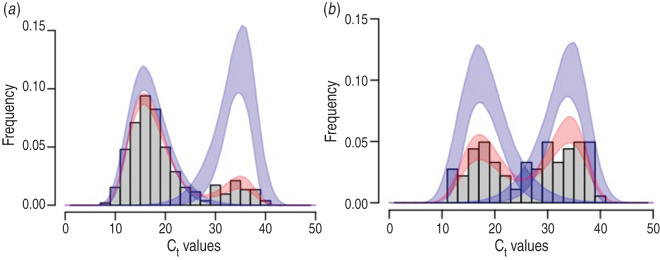
Log-normal and Weibull mixture model of norovirus C_t_ values (same as Fig. 1) applied separately to (*a*) rotavirus (RV)-negative samples (*n* = 259) and (*b*) RV-positive samples (*n* = 87). Distributions *D*_1_ and *D*_2_ were log-normal and Weibull, respectively (Supplementary Tables S2 and S3).

The typical bimodal distribution of NV C_t_ values (Figs 1 and 2) was modelled by a finite-mixture model using continuous unimodal distributions from the exponential family [28]. In the absence of prior information on expected distribution for each peak, we evaluated the normal, log-normal, gamma, Weibull distributions, and all possible combinations (4 × 4 = 16). The normal distribution is defined on all the real numbers, whereas the three other distributions (log-normal, Weibull, gamma) are defined on positive reals. These distributions are all characterized by two parameters: a location parameter *μ* and a scale parameter *σ*. The first parameter accounts for most of the data and corresponds to the mean of the normal distribution, the mean on a log-scale for the log-normal distribution, and the shape parameter for the gamma and Weibull distributions. The second parameter accounts for the spread of the data around the location parameter and corresponds to the standard deviation in the case of the normal distribution, the standard deviation on a log-scale for the log-normal distribution, the rate parameter (1/scale) for the gamma distribution and the scale parameter for the Weibull distribution. We refer to *μ*_1_ and *σ*_1_ for the location and scale parameters of the first peak (i.e. lowest C_t_ values) and *μ*_2_ and *σ*_2_ for the location and scale parameters of the second peak (i.e. highest C_t_ values). The density of the bimodal distribution of C_t_ values thus reads

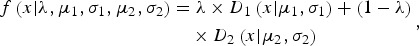

where *D*_1_ and *D*_2_ are the distributions accounting for the first (i.e. lowest C_t_ values) and second (i.e. the highest C_t_ values) peaks, respectively, and *λ* and (1 – *λ*) are the weights for the *D*_1_ and *D*_2_ distributions, respectively.

For each of the 16 combinations of distributions, parameters *λ, μ*_1_, *σ*_1_, *μ*_2_, *σ*_2_ were estimated by maximum likelihood (ML) using the expectation-maximization (EM) algorithm [29], the confidence intervals were calculated as proposed by Oakes [30] and all calculations were done in R [31]. Given the equal numbers of parameters (*n* = 5) of the 16 models, the best combination of distributions for the two peaks was chosen as the one minimizing the minus log-likelihood. From parameterized distribution *D*_1_ and *D*_2_ of the best model, we computed the probability *P* to belong to the low C_t_ value peak as *P* = *D*_1_/(*D*_1_ + *D*_2_) and this probability was used to derive a cut-off value separating the two peaks.

### Antigen detection of NV and RV by ELISA

RV antigens were tested on all samples whereas NV antigens were tested in a subset of samples (*n* = 182). Of these 182 samples, 47 were randomly selected from the NV-negative samples (C_t_ ⩾40) and 135 NV-positive samples were randomly selected to represent a uniform distribution of C_t_ values <40. NV and RV antigen detection was performed by NV-AD-III kit (Denka Seika, Japan) and Rotaclone™ enzyme immunoassay (Meridian, USA) according to the manufacturers' instructions, respectively. The cut-off for NV and RV positivity was OD_450nm_ = 0·15; samples within 0·01 OD of the cut-off were repeated to confirm results.

## RESULTS

### Characteristics of children admitted to Thai Binh paediatric hospital

From January 2011 to September 2012, a total of 807 faecal samples were collected and examined for the presence of NV and RV. In Thai Binh Paediatric hospital, 89% and 97% of children hospitalized for diarrhoea were aged less than 24 and 36 months, respectively (Table 1). NV and RV were identified in 43% and 40% of children with diarrhoea, respectively; co-infections with both viruses occurred in 87 (11%) patients, and 227 (28%) were negative for both NV and RV.

**Table 1.** Age distribution and molecular screening results for rotavirus (RV) and norovirus (NV) in patients hospitalized with diarrhoea in Thai Binh, Vietnam, January 2011–September 2012

Age group (months)	Total	NV* C_t_ cut-off = 40	NV* C_t_ cut-off = 21·36	RV*	RV-NV co-infection
	*N* (%)	*n* (%)	*n* (%)	*n* (%)	*n* (%)
0–2	26 (3·2)	4 (1·2)	2 (0·9)	5 (1·6)	0
3–5	126 (15·6)	53 (15)	31 (14)	36 (11)	13 (15)
6–11	327 (40·5)	148 (43)	105 (47)	126 (39)	33 (38)
12–23	243 (30)	111 (32)	67 (30)	118 (37)	34 (39)
24–35	58 (7·2)	22 (6·4)	13 (5·8)	26 (8·1)	6 (7)
36–47	18 (2·2)	6 (1·7)	4 (1·8)	7 (2·2)	1 (1·1)
48–60	9 (1·1)	2 (0·6)	1 (0·4)	3 (0·9)	0
Total	807 (100)	346 (100)	223 (100)	321 (100)	87

* Including both cases of single infection and co-infection.

A total of 285 NV samples (92% of total samples with C_t_ <35) were genotyped. NV-GII was the dominant genogroup (91%); GII.3 and GII.4 genotypes constituted 27% and 59%, respectively. Other genotypes of NV-GII included GII.2 (0·6%), GII.7 (0·3%), GII.12 (0·3%), GII.13 (2·6%) and GII.16 (0·6%). Only three (0·9%) cases of GI.8 were identified, one of which was a co-infection with GII.3.

### Cut-off C_t_ value for NV-associated diarrhoea

The 16 finite-mixture models were fitted to the 346 C_t_ values <40 (represented by the grey histogram in Fig. 1). According to the log-likelihood, the best fit model comprised a log-normal distribution for *D*_1_ and Weibull distribution for *D*_2_, with *λ* = 74% [95% confidence interval (CI), 70–79] for the values belonging to the lower peak of C_t_ values (Supplementary Table S1, Fig. 1). The constitutive distributions *D*_1_ and *D*_2_ are the left-most and right-most curves, respectively, from which the probability *P* of belonging to the lower peak C_t_ values peak is represented by the green curve. From this latter we infer that a probability *P* of 50% belongs to the lower peak of C_t_ values, which corresponds to a cut-off of 25·45 (95% CI 25·45–25·45); a probability *P* of 95% belongs to the lower peak of C_t_ values corresponding to a cut-off C_t_ of 21·36 (95% CI 20·29–22·46); a probability *P* of 99% belongs to the higher peak of C_t_ values corresponding to a cut-off C_t_ of 19·01 (95% CI 17·59–20·45). Using the 95% *P* C_t_ cut-off (21·36), the adjusted NV detection rates reduced significantly from 43% (95% CI 41–51) to 28% (95% CI 29–38) (Table 1). Thus, using these new criteria, RV and NV were causative agents in 40% and 28% of diarrhoea cases, respectively.

### Co-infection with RV

Of the 346 patients positive for NV by real time RT-PCR, 87 were ELISA-positive for RV, and 259 were ELISA-negative for RV. When comparing RV positive and negative cases, we found no significant differences in age (Welch's two-sample *t* test: *t* = −0·4389, d.f. = 104·20, *P* = 0·66, Mann–Whitney two-sample test: *W* = 6424·50, *P* = 0·82), gender (Fisher's exact test: *P* = 0·64), NV genotype composition (Fisher's exact test: *P* = 0·85) or duration between diarrhoea onset and sampling (Welch's two-sample *t* test: *t* = 0·62, d.f. = 76·22, *P* = 0·54; Mann–Whitney two-sample test: *W* = 6468, *P* = 0·75). When fitting the same finite-mixture models separately to the 87 ELISA-RV-positive and the 259 ELISA-RV-negative patients, the best-fit model had a log-normal distribution for *D*_1_ and a Weibull distribution for *D*_2_ (Fig. 2, Supplementary Tables S2, S3). As expected, the proportion *λ* of C_t_ values belonging to the lower peak of C_t_ values was significantly higher in RV-negative patients (84%, 95% CI 80–89) than in RV-positive patients (44%, 95% CI 35–55).

### Effects of host, virus genotypes and time elapsed between diarrhoea onset and sampling

Patients' age did not differ significantly between the two peaks of C_t_ values (Welch's two-sample *t* test: *t* = −0·80, d.f. = 142·80, *P* = 0·43; Mann–Whitney two-sample test: *W* = 11 953·5, *P* = 0·96, see Supplementary Fig. S1A). The proportion of genotype GII.4 (Supplementary Fig. S1D; *χ*^2^ = 0·32, d.f. = 279, *P* = 0·57), and the proportion of males and of genotype GII.3 marginally significantly increased as the C_t_ values decreased: *χ*^2^ = 3·34, d.f. = 279, *P* = 0·07 and *χ*^2^ = 3·17, d.f. = 344, *P* = 0·08 (see Supplementary Fig. S1C), respectively. By contrast, the time-lag between diarrhoea onset and sampling marginally significantly increased with C_t_ values (Welch's two-sample *t* test: *t* = −1·78, d.f. = 153·69, *P* = 0·08; Mann–Whitney two-sample test: *W* = 10 383·50, *P* = 0·05) (see Supplementary Fig. S1B).

### Comparison of NV antigen ELISA and RT–PCR cut-off values

All 47 samples with NV C_t_ ⩾40 were negative by NV ELISA. Of the 135 samples with NV C_t_ <40, 72 samples were positive by NV ELISA; 70/72 (97%) antigen-positives had C_t_ <21·36; and only 2/53 (4%) samples with C_t_ >21·36 were NV antigen-positive (Supplementary Fig. S2). Of the samples with C_t_ <21·36, only 70/82 (85%) were antigen positive. The genotypes of samples with high viral load (C_t_ <21·36) that were negative by antigen ELISA were GII.3 (5/28), GII.4 New Orleans 2009 (*n* = 3/3), GII.4 2006b (2/42) and GII.13 (1/1). Determination of specificity and sensitivity of the NV antigen ELISA test (Supplementary Table S4) showed 98% specificity (96% if considering only C_t_ <40) and 85% sensitivity for samples belonging to the lower peak of C_t_ values (*P* = 0·95).

## DISCUSSION

Several previous studies have evaluated NV viral loads in paediatric populations in order to better understand dynamics of NV shedding in diarrhoeal cases *vs.* healthy controls [19, 32]. Barreira *et al*. [19] reported tenfold higher NV RNA copy numbers in diarrhoeal cases compared to controls, and Phillips *et al.* [32] observed the range of C_t_ values in children aged <5 years with diarrhoea [interquartile range (IQR) 32–37, median 35] and in age-matched healthy/non-diarrhoeal controls (IQR 34–38, median 37), although these groups overlapped substantially. Phillips *et al*. used the Youden index and ROC curve analysis of diarrhoeal and healthy control groups to propose a C_t_ cut-off value for NV gastroenteritis; they suggested a C_t_ <30 for children aged <5 years, and C_t_ = 33 for older children and adults. Recently, Elfving *et al.* presented a study to determine threshold cycle cut-offs for multiple pathogens causing acute diarrhoea [33]. The study proposed cut-off values for *Cryptosporidium, Shigella*, and ETEC-estA (35, 30, 31, respectively); however, no value could be identified for RV and NV [33].

Here we propose an analytical approach to distinguish diarrhoeal cases in which NV is likely to have played a causative role in disease presentation *vs.* cases in which an alternative pathogen may be involved. Our method was based on the distribution curves of NV C_t_ values, and did not require comparison to healthy controls. It was founded on the observations that (i) the distribution of C_t_ values for NV is generally bimodal, (ii) NV RNA in healthy children are detected at similar or even higher rates than in diarrhoea cases [12, 19]; however, (iii) diarrhoea cases shed larger amount of NV than healthy children [19]. We modelled the bimodal distribution of C_t_ values with a finite-mixture model, and tested the hypothesis that the lower peak of C_t_ values corresponded to samples for which the cause of diarrhoea was NV and the higher peak corresponded to samples where other agents were involved. Our working hypothesis was tested by applying our method to a subset of samples with RV co-infections, and by evaluating confirmatory NV diagnoses using NV antigen ELISA. As expected, the proportion of samples with low NV viral load had significantly more RV co-infections, whereas those with high NV viral loads were more likely to be RV negative. By contrast, the interval between onset of diarrhoea and sampling, patient gender, and virus genotypes were not significant in explaining NV viral load.

After applying the cut-off value obtained in this study to our own dataset, the proportion of NV cases decreased from 43% (C_t_ <40) to 28% (C_t_ <21·36). However, there was no change in the age distribution of cases. Similar NV prevalence (20·6%) was found in children with diarrhoea in a study conducted in Ho Chi Minh city during 2009–2010 [10]. Due to the complicated nature of NV infections, interpretations of low viral loads are difficult, as these values may still indicate a causative role in symptomatic infections; indeed, the authors suggest that clinical diagnostic laboratories must evaluate appropriate cut-off values for different patient populations.

The probability of positive NV ELISA test increases when the C_t_ value decreases and this can be explained by the relatively poor sensitivity of antigen detection by ELISA compared to RT-PCR. When comparing our method with ELISA, our proposed C_t_ <21·36 cut-off agreed well with ELISA, with 98% specificity (96% when considering only the C_t_ <40). The observation that some NV genotypes and variants were not detected by ELISA may explain the lower sensitivity (85%) of ELISA. Effectively, this means that a positive NV ELISA test can be considered as indicative of a case where NV is the actual cause of the diarrhoea, whereas only 90% (81% if considering only C_t_ <40) of negative NV ELISA tests can be considered as indicative of a diarrhoea case not caused by NV. It is worth noting that the C_t_ <21·36 threshold is in the range reported by Costantini *et al.* (IQR 16·5–22·9, median 19·1) in which most samples were positive by ELISA [34]. Costantini *et al.* suggested that if samples are collected 48 h after onset of the symptoms, ELISA may yield false negatives.

Due to differences in laboratory practices, it is not feasible to directly compare our suggested cut-off of C_t_ <21·36 to the value of C_t_ = 31 proposed by Phillips *et al*. [32] for distinguishing cases from controls. Of note, our study involved exclusively hospitalized cases of diarrhoea, whereas in the Phillips *et al.* study cases were recruited from primary care and the community, thus representing a broader spectrum of disease severity. In addition to differences in study design, different sampling techniques, sample quality issues, RNA extraction methods and sample preparation procedures are likely to affect virus genome concentrations, while different RT–PCR assays may vary in sensitivity. We suggest that each laboratory should conduct its own analysis to evaluate distribution curves and determine an appropriate cut-off value for NV-associated disease. The advantage of our approach is that such determinations of a C_t_ threshold for NV-associated disease may be generated in the absence of data from controls, and thus, could be applied to any laboratory receiving clinical samples on a regular basis.

In conclusion, we propose a threshold C_t_ value for real-time RT–PCR to assess the aetiological role of NV in children with diarrhoea. Our statistical method allowed determination of a cut-off value without reference to any controls, which is essential for the feasibility of extending this analysis to other laboratories conducting routine epidemiological surveillance and clinical diagnostics. We suggest that cut-off values should be determined individually in each laboratory based on its own assay performance, and that accumulation of these types of data across multiple laboratories would contribute to improved understanding of the burden of NV disease.

## Supplementary material

For supplementary material accompanying this paper visit https://doi.org/10.1017/S095026881500059X. click here to view supplementary material
