# Supplementary material for: Determination of cut-off cycle threshold values in routine RT–PCR assays to assist differential diagnosis of norovirus in children hospitalized for acute gastroenteritis
Source: Epidemiol Infect. 2015 Apr 1;143(15):3292–9. doi: 10.1017/S095026881500059X (PMC4594052; doi:10.1017/S095026881500059X)
Supplement: Supplementary file 1 [file S095026881500059Xsup001.zip › Supplementary Figure legends.docx]

**Fig. S1**. Impact of patients' age (A), sampling delay (days post onset) (B), gender (C), and virus genotype (D) on C_t_ value. The vertical dotted line shows the C_t_ threshold value of 25.45 corresponding to a probability of 0.50 of belonging to the first peak. Grey dots show the data points. The boxes indicate the interquartile ranges (IQR) between first and third quartiles, and horizontal lines indicate the median. Whiskers are the lowest and highest values within 1.5 IQR, with outliers shown by black points. The widths of the boxes are proportional to the number of data points. (B): rugs show the males (top) and females (bottom). (B-C): Lines and grey areas show logistic models and their 95% CI. Dots are the means with 95% CI intervals for the two peaks separately.

**Fig. S2.** Probability of NV-ELISA positive test (grey) from a logistic model compared to the probability of belonging to the second peak (green). Rugs show the NV-ELISA positive (top) and negative (bottom) samples and the areas show the 95% CI. The vertical dashed line indicates the C_t_ threshold 21.36 corresponding to a probability *P* = 0.95 of belonging to the first peak.
