# Supplementary figures and images for: Determination of cut-off cycle threshold values in routine RT–PCR assays to assist differential diagnosis of norovirus in children hospitalized for acute gastroenteritis
Source: Epidemiol Infect. 2015 Apr 1;143(15):3292–9. doi: 10.1017/S095026881500059X (PMC4594052; doi:10.1017/S095026881500059X)

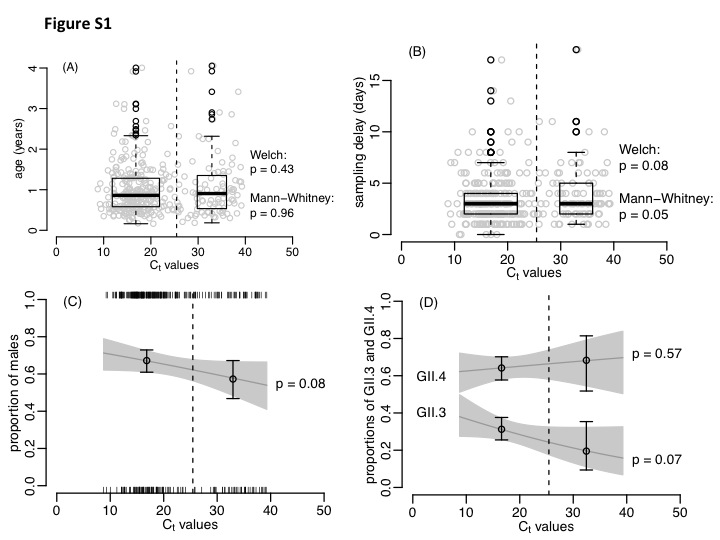

Supplement: Supplementary file 1 [file S095026881500059Xsup001.zip › FigureS1.jpg]

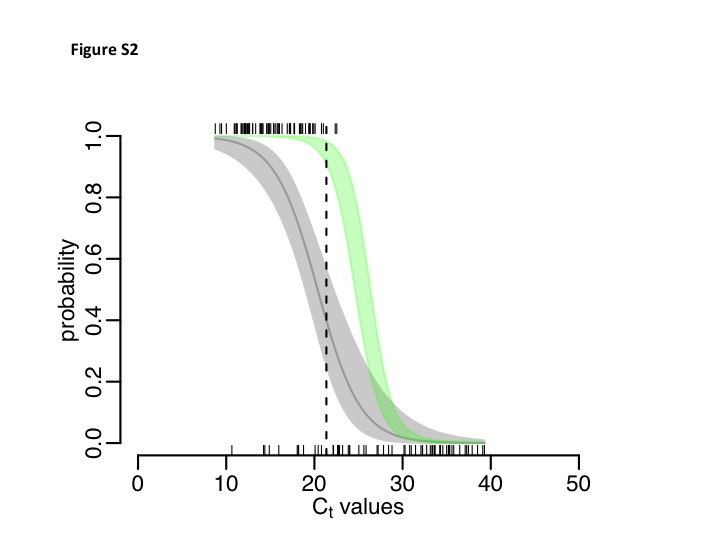

Supplement: Supplementary file 1 [file S095026881500059Xsup001.zip › FigureS2.jpg]
