# Supplementary material for: Determination of cut-off cycle threshold values in routine RT–PCR assays to assist differential diagnosis of norovirus in children hospitalized for acute gastroenteritis
Source: Epidemiol Infect. 2015 Apr 1;143(15):3292–9. doi: 10.1017/S095026881500059X (PMC4594052; doi:10.1017/S095026881500059X)
Supplement: Supplementary file 1 [file S095026881500059Xsup001.zip › Trang-EpiInf-tableS1-SUBMIT-final.doc]

Table S1: Results of the maximum-likelihood expectation-maximum algorithm to fit the 16 possible finite mixture models to the 346 NV Ct values below 40 by the histogram on Figure 1. µ_1_ and σ_1_ are the parameters of the distribution D_1_ and µ_2_ and 2 are the parameters in distribution D_2_.  is the weight of the distributions D_1_ in finite mixture model and –LL is the minus log-likelihood of the fitted model. Numbers in brackets indicate the 95% confidence intervals.

|  | D_1_ | D_2_ | µ_1_ | σ_1_ | µ_2_ | σ_2_ | -LL_min_ | λ (95%CI) |
| --- | --- | --- | --- | --- | --- | --- | --- | --- |
| 1 | normal | normal | 16.81 | 3.50 | 32.76 | 4.06 | 1148.41 | 0.71 (0.67, 0.76) |
| 2 | normal | log-normal | 16.97 | 3.65 | 3.50 | 0.11 | 1151.34 | 0.73 (0.68, 0.77) |
| 3 | normal | gamma | 16.91 | 3.59 | 71.87 | 2.18 | 1150.31 | 0.72 (0.68, 077) |
| 4 | normal | Weibull | 16.69 | 3.41 | 8.86 | 34.19 | 1144.39 | 0.70 (0.65, 0.75) |
| 5 | log-normal | normal | 2.83 | 0.24 | 33.82 | 3.25 | 1143.23 | 0.76 (0.71, 0.80) |
| 6 | log-normal | log-normal | 2.83 | 0.24 | 3.52 | 0.09 | 1144.61 | 0.76 (0.72, 0.81) |
| 7 | log-normal | gamma | 2.83 | 0.24 | 113.91 | 3.36 | 1144.12 | 0.76 (0.72, 0.81) |
| 8 | ***log-normal*** | ***Weibull*** | ***2.83*** | ***0.23*** | ***10.93*** | ***34.96*** | ***1140.70*** | ***0.74 (0.70, 0.79)*** |
| 9 | gamma | normal | 19.56 | 1.14 | 33.56 | 3.44 | 1144.09 | 0.74 (0.70, 0.79) |
| 10 | gamma | log-normal | 18.87 | 1.09 | 3.52 | 0.10 | 1145.79 | 0.75 (0.71, 0.80) |
| 11 | gamma | gamma | 19.08 | 1.10 | 102.65 | 3.04 | 1145.20 | 0.75 (0.70, 0.79) |
| 12 | gamma | Weibull | 20.70 | 1.22 | 10.23 | 34.72 | 1141.10 | 0.73 (0.68, 0.77) |
| 13 | Weibull | normal | 5.55 | 17.89 | 32.10 | 4.55 | 1153.92 | 0.69 (0.64, 0.74) |
| 14 | Weibull | log-normal | 5.32 | 18.11 | 3.48 | 0.13 | 1157.61 | 0.71 (0.66, 0.75) |
| 15 | Weibull | gamma | 5.41 | 18.03 | 55.26 | 1.70 | 1156.29 | 0.70 (0.65, 0.75) |
| 16 | Weibull | Weibull | 5.63 | 17.79 | 7.94 | 33.74 | 1149.57 | 0.68 (0.63, 0.73) |
