# Supplementary material for: Determination of cut-off cycle threshold values in routine RT–PCR assays to assist differential diagnosis of norovirus in children hospitalized for acute gastroenteritis
Source: Epidemiol Infect. 2015 Apr 1;143(15):3292–9. doi: 10.1017/S095026881500059X (PMC4594052; doi:10.1017/S095026881500059X)
Supplement: Supplementary file 1 [file S095026881500059Xsup001.zip › Trang-EpiInf-tableS2-SUBMIT-final.doc]

Table S2. Parameter estimates for 16 mixture models using 87 samples with NV Ct value <40 and positive for RV by ELISA. Refer to text for parameter definitions

| STT | D_1_ | D_1_ | µ_1_ | σ_1_ | µ_2_ | σ_2_ | -LL_min_ | λ |
| --- | --- | --- | --- | --- | --- | --- | --- | --- |
| 1 | normal | normal | 17.95 | 3.68 | 32.88 | 3.87 | 305.73 | 0.43 (0.33, 0.54) |
| 2 | normal | log-normal | 18.58 | 4.15 | 3.50 | 0.11 | 306.92 | 0.47 (0.37, 0.57) |
| 3 | normal | gamma | 18.35 | 3.98 | 82.80 | 2.49 | 306.52 | 0.46 (0.36, 0.56) |
| 4 | normal | Weibull | 17.48 | 3.35 | 9.05 | 34.18 | 303.92 | 0.40 (0.30, 0.50) |
| 5 | log-normal | normal | 2.92 | 0.24 | 33.46 | 3.45 | 304.47 | 0.49 (0.39, 0.60) |
| 6 | log-normal | log-normal | 2.94 | 0.25 | 3.51 | 0.10 | 305.3 | 0.51 (0.42, 0.62) |
| 7 | log-normal | gamma | 2.93 | 0.25 | 102.68 | 3.05 | 305.01 | 0.51 (0.40, 0.61) |
| ***8*** | ***log-normal*** | ***Weibull*** | ***2.88*** | ***0.22*** | ***10.03*** | ***34.58*** | ***303.02*** | **0.44 (0.35, 0.55)** |
| 9 | Gamma | normal | 19.15 | 1.03 | 33.32 | 3.55 | 304.8 | 0.47 (0.37, 0.58 |
| 10 | Gamma | log-normal | 16.99 | 0.89 | 3.51 | 0.10 | 305.69 | 0.50 (0.40, 0.61) |
| 11 | Gamma | gamma | 17.62 | 0.93 | 98.47 | 2.93 | 305.38 | 0.49 (0.39, 0.60) |
| 12 | Gamma | Weibull | 22.56 | 1.25 | 9.70 | 34.45 | 303.25 | 0.43 (0.33, 0.53) |
| 13 | Weibull | normal | 5.53 | 19.13 | 32.70 | 4.00 | 306.72 | 0.42 (0.32, 0.52) |
| 14 | Weibull | log-normal | 4.82 | 20.29 | 3.50 | 0.10 | 307.83 | 0.47 (0.37, 0.58) |
| 15 | Weibull | gamma | 4.94 | 20.04 | 86.86 | 2.61 | 307.47 | 0.46 (0.36, 0.57) |
| 16 | Weibull | Weibull | 6.07 | 18.51 | 8.66 | 34.02 | 304.78 | 0.38 (0.28, 0.48) |
