# Supplementary material for: Determination of cut-off cycle threshold values in routine RT–PCR assays to assist differential diagnosis of norovirus in children hospitalized for acute gastroenteritis
Source: Epidemiol Infect. 2015 Apr 1;143(15):3292–9. doi: 10.1017/S095026881500059X (PMC4594052; doi:10.1017/S095026881500059X)
Supplement: Supplementary file 1 [file S095026881500059Xsup001.zip › Trang-EpiInf-tableS3-SUBMIT-final.doc]

Table S3. Parameter estimates for 16 mixture models using 259 samples with NV Ct value <40 and negative for RV by ELISA. Refer to text for parameter definitions.

| STT | D_1_ | D_2_ | µ_1_ | σ_1_ | µ_2_ | σ_2_ | -LL_min_ | λ |
| --- | --- | --- | --- | --- | --- | --- | --- | --- |
| 1 | normal | normal | 16.65 | 3.48 | 32.87 | 4.08 | 817.72 | 0.82 (0.77, 0.86) |
| 2 | normal | log-normal | 16.77 | 3.60 | 3.50 | 0.11 | 819.19 | 0.83 (0.78, 0.87) |
| 3 | normal | gamma | 16.73 | 3.55 | 73.77 | 2.22 | 818.68 | 0.82 (0.78, 0.87) |
| 4 | normal | Weibull | 16.58 | 3.43 | 8.91 | 34.30 | 815.65 | 0.81 (0.76, 0.86) |
| 5 | log-normal | normal | 2.81 | 0.23 | 34.15 | 3.08 | 813.82 | 0.85 (0.81, 0.89) |
| 6 | log-normal | log-normal | 2.81 | 0.23 | 3.53 | 0.09 | 814.39 | 0.85 (0.81, 0.90) |
| 7 | log-normal | gamma | 2.81 | 0.23 | 127.32 | 3.72 | 814.19 | 0.85 (0.81, 0.89) |
| ***8*** | ***log-normal*** | ***Weibull*** | ***2.81*** | ***0.23*** | ***11.73*** | ***35.27*** | ***812.74*** | ***0.84 (0.80, 0.89)*** |
| 9 | gamma | normal | 20.03 | 1.18 | 33.87 | 3.27 | 814.22 | 0.84 (0.80, 0.89) |
| 10 | gamma | log-normal | 19.68 | 1.16 | 3.52 | 0.09 | 814.93 | 0.84 (0.80, 0.89) |
| 11 | gamma | gamma | 19.78 | 1.17 | 114.02 | 3.35 | 814.68 | 0.84 (0.80, 0.89) |
| 12 | gamma | Weibull | 20.76 | 1.23 | 10.83 | 34.99 | 812.85 | 0.83 (0.78, 0.88) |
| 13 | Weibull | normal | 5.60 | 17.66 | 31.44 | 5.07 | 822.36 | 0.78 (0.73, 0.83) |
| 14 | Weibull | log-normal | 5.42 | 17.83 | 3.46 | 0.15 | 824.42 | 0.80 (0.75, 0.85) |
| 15 | Weibull | gamma | 5.49 | 17.77 | 44.02 | 1.38 | 823.67 | 0.79 (0.74, 0.84) |
| 16 | Weibull | Weibull | 5.56 | 17.68 | 7.37 | 33.49 | 820.25 | 0.78 (0.73, 0.83) |
