# Supplementary material for: Determination of cut-off cycle threshold values in routine RT–PCR assays to assist differential diagnosis of norovirus in children hospitalized for acute gastroenteritis
Source: Epidemiol Infect. 2015 Apr 1;143(15):3292–9. doi: 10.1017/S095026881500059X (PMC4594052; doi:10.1017/S095026881500059X)
Supplement: Supplementary file 1 [file S095026881500059Xsup001.zip › Trang-EpiInf-tableS4-SUBMIT-final.doc]

Table S4. Comparison of ELISA and NV molecular screening results.

|  | NV-ELISA positive | NV-ELISA negative |
| --- | --- | --- |
| NV-Ct < 21.36 | 70 | 12 |
| NV-Ct > 21.36 | 2 | 51+56* |

*These 56 correspond to the expected additional number of samples with Ct values above 40. Among the 807 samples of the study, 43% had Ct<40. However, among the samples tested for ELISA, 74% (135 out of 182) had a Ct<40. Furthermore, all 47 samples with Ct>40 were ELISA negative. Thus, to generalize ELISA results obtained on samples with Ct<40 to all samples, we add (70+12-0.43*(70+12+2+51))/0.43 = 56 samples with Ct>40.
